# Diagnostic Prospectives with Tau Protein and Imaging Techniques to Detect Development of Chronic Traumatic Encephalopathy

**DOI:** 10.4236/jbbs.2023.134005

**Published:** 2023-04-30

**Authors:** Amit Naskar, Danielle Jayanty, Kimberly Head, Gulshan L. Khanna, Vatsalya Vatsalya, Arpan Banerjee

**Affiliations:** 1 Cognitive Brain Dynamics Lab, National Brain Research Centre, Manesar, India; 2 Department of Medicine, University of Louisville, Louisville, KY, USA; 3 Professor and Pro Vice Chancellor, Manav Rachna International Institute of Research and Studies, Faridabad, India; 4 Robley Rex VA Medical Center, Louisville, KY, USA; 5 National Institute on Alcohol Abuse and Alcoholism, NIH, Bethesda, MD, USA

**Keywords:** Athletes, Axonal Injury, Tau Protein, Chronic Traumatic Encephalopathy, Traumatic Brain Injury, Boxing, Wrestling

## Abstract

Brain damage sustained from repeated blows in boxing, wrestling, and other combat sports has serious physical and mental health consequences. The degenerative brain disease, chronic traumatic encephalopathy (CTE), presents clinically with memory loss, aggression, difficulty in rational thinking and other cognitive problems. This spectrum, which mimics Alzheimer’s disease, is diagnosed post-mortem through a brain biopsy in many professional athletes. However, little is known about the process of development and how to identify vulnerable individuals who may be on course for developing CTE. Boxing is a sport that has a severe toll on athletes’ health, primarily on their brain health and function. This review addresses the concerns of brain injury, describes the pathologies that manifest in multiple scales, e.g., molecular and cognitive, and also proposes possible diagnostic and prognostic markers to characterize the early onset of CTE along with the aim to identify a starting point for future precautions and interventions.

## 1. Background

Concussion is characterized as a mild traumatic brain injury, which is a known risk factor for CTE in contact sports. Early detection and intervention with a period of rest are the primary standard of care (medical management) strategies to alleviate the long-term and short-term effects of concussion. Although many athletes involved in boxing suffer concussions (a traumatic brain injury that affects brain function remarkably) frequently, the number who undergo repeated head hits that do not result in concussion is even higher. These subconcussive head hits (SHH) are especially prevalent during their training period as well as the amateur career [1]. SHH are the impact on the head (such as a bump, blow, or a jolt that does not cause any symptoms) have been linked to long-term problems with memory, depression, and possibly a form of dementia called chronic traumatic encephalopathy (CTE) [2]. SHH may have cumulative adverse effects that may lead to neurological dysfunction in later life [3]. Unlike concussions that can be treated with rest; SHH is difficult to detect and is not currently recognized as a clinically significant condition. The lack of fully characterized clinical presentation limits the treatment of SHH [4]. SHH represents an ignored but potentially treatable condition that can impact the long-term neurologic health of many athletes who participate in contact sports, especially in developing regions of the world e.g., India, where imaging-based medical care is not routinely performed.

The clinical presentation of a concussion is comprised of specific medical signs and symptoms which present at the time of injury [5]. This presentation constitutes an immediate and transient posttraumatic impairment of neural function. For example, there is an alteration of consciousness or disturbance of vision or equilibrium arising from the involvement of brain stem pathology [6]. There are treatments and guidelines regarding contact sports that are available for athletes after a cerebral concussion [7]. However, mechanisms involved in brain injury due to SHH have been studied sparingly, and thus clear clinical presentations and interventions are not well defined. SHH have recently been investigated in football players to predict altered brain metabolism in the undiagnosed injured population [8]. These findings validated a previous investigation that identified damaged glial cells, membrane turnover, disrupted energy metabolism, and neurotransmission in non-diagnosed collision sport athletes [9]. This kind of traumatic brain injury is subtle in clinical presentation even if it amounts to a noticeable deviant state of function and structural integrity [10]. During the training period, especially in adolescent athletes, such collisions could be harmful to developmental morphology, and can have damaging adverse effects on the functional growth of the developing brain [11]. Within traumatic brain injury research, there is a need for exploring powerful new technologies that measure brain function, which could inform effective training protocols targeted toward allaying SHH-induced injuries.

Traumatic brain injury (TBI) is considered a silent epidemic among head injuries and there have been mostly region-specific initiatives taken to address the pathology and harmful effects on athletes in developed countries during the early 1990s [12]. CTE is a type of TBI that is also considered as an independent neurodegenerative disorder characterized by perivascular deposits of hyperphosphorylated tau at the depths of the cerebral sulci [13]. Boxing and similar combat sports result in repetitive head trauma that could lead to CTE causing brain damage and cognitive deficits [14]. Studies worldwide have reported serious brain injury not only in professionals but also in amateur boxers; and evidence for CTE in boxing is widely accepted [15]. SHH could contribute to CTE over a period of time [16]. Clinical features of CTE and its association with the degree of exposure have shown relevance with radiological imaging. One important investigational area is how many athletes are affected by CTE and how to critically define the condition. While one study showed that 17% of boxers receiving head hits could be diagnosed with CTE, another reported that around 40% of the remaining boxers had some kind of neurodegeneration, or drug addiction (or withdrawal including alcoholism) to reduce pain from head injuries related to boxing [17]. SHH could inflict long-term consequences that could manifest as mental, cognitive, and neurological conditions [13]. Studies on TBI in the last decade have revealed that CTE occurs not only in boxers but also in a wider population including American football players, and wrestlers where repetitive head injury is prevalent [18].

Recent studies in neurodegenerative disease brain bank cohorts suggest that changes in CTE are relatively common [19]. The clinical presentation of CTE characteristically begins in one or more of the four distinct neurological domains: mood, behavior, cognition, and motor (Stern *et al.*, 2013). Few reports described early behavioral signs as explosivity, verbal and physical violence, loss of control, impulsivity, paranoia and rage behaviors [20] [21]. Cognitively, the most prominent deficits are memory, executive functioning, and impaired attention. Approximately 45% of subjects with CTE develop dementia. Complaints of chronic headaches occur in 30% [20] of those suffering from CTE. Motor symptoms could include dysarthria, dysphagia, coordination problems, and Parkinsonism (tremors, decreased facial expression, rigidity, and gait instability) that may develop during the early course or once a diagnosis is established [22].

Overall, there are significant overlaps in neuro-pathological features between CTE and other neurodegenerative disorders, e.g., chronic neuro-inflammation, widespread astrogliosis and microgliosis. Deposition of neurofilament phospho-tau tangles has already been reported in CTE (Falcon *et al.*, 2019). Tau protein, which is a biomarker observed in brain injury, can be seen in various neuropathologies and could also be useful in predicting the extent and severity of brain injury and damage in SHH. In this review, we discuss the scanning techniques and relevance of TAU protein.

## 2. Pathology and Biomarkers of Brain Injury

Blood-based brain biomarkers (BBBM) for example S100B, GFAP, and tau have been reported to address and describe traumatic axonal injury (AI) [23] [24]. They have emerged as tools to detect concussions early in the pathological course [25]. However, repetitive head hits that do not result in concussion could also be a potential threat to long-term neurologic health. These SHHs have been linked to a spectrum of clinical presentations such as the increased risk of long-term cognitive dysfunction, depression, and CTE [26]. Unlike concussions, SHH that could result in AI are not routinely identified and thus are not treated with a period of rest without head hits [27]. This condition could arise during the training period and initial stages of competitive events. BBBMs could detect AI-producing SHH [23] but have not been specifically studied in this regard. These AI-producing SHH represent a potentially treatable threat to the long-term neurologic health of athletes involved in contact sports. Thus, it would be important to investigate if the BBBM, such as tau, could detect AI-producing SHHs incurred during a single sporting contest to establish a baseline and further understand SHH in the context of developing CTE. Directly developing BBBM as a tool for studying the natural history of SHHs and their impact on long-term brain health would be highly beneficial to estimate ongoing and cumulative brain injury.

Although there is no single diagnostic biomarker currently available to characterize and define the pathology of brain injury for athletes involved in combat sports, several promising techniques are being developed. The use of *in-vivo* biomarkers could greatly improve the accurate clinical diagnosis of CTE and facilitate in the monitoring of disease progression and the efficacy of disease-modifying therapies. Tau-specific PET ligands have demonstrated encouraging results in Alzheimer’s disease (AD) [28] [29] and detected the progression of AD tauopathy among individuals along the cognitive spectrum [30]. Studies using diffusion tensor imaging have also shown promise in their ability to detect changes to white matter (WM) integrity following head trauma [31]. In addition, functional connectivity obtained from fMRI scans and other advanced imaging measures of brain metabolites, such as magnetic resonance spectroscopy is useful in detecting the biochemical changes, cerebrospinal fluid, and plasma protein markers (including p-tau and total tau) [32] [33].

Contact sports like boxing have become popular worldwide and in boxing the athletes receive sub-concussive head hits that may not show any symptoms right away, but we do not know how long and how much exposure is needed to cause CTE. The burden of the disease will continue to increase without further understanding of inciting events like SHHs in the development of CTE. Changes in the cytokine response due to injury and inflammation, pathological changes in brain regions, associated movement disorders, and importantly targeted intervention are crucial aspects of identifying progressive abnormalities [34] [35] [36]. These concerns are targeted toward detecting and staging brain abnormalities. The absence of proper guidelines limits individuals to decide between continuing the sport or abstaining from training and competitive events. This current model perpetuates the aggravation of the disease because athletes and their physicians cannot make informed decisions.

Thus there are gaps that limit the understanding of brain injury due to SHH: 1) changes that take place in and around the damaged area of the brain immediately after injury; 2) ways of mitigating further damage by preventing, impeding, or reversing the progression of ischemic and other destructive biochemical and metabolic changes; 3) understanding the mechanisms of brain repair or compensation as a step towards the restitution of function; and 4) restoration of function to victims of head injury. Future studies are necessary to elucidate the mechanisms of neurodegeneration in athletes with CTE.

Oneneuropathological study found that CTE demonstrates a unique pattern of tau pathology in neurons and astrocytes [37]. Development in neuroimaging techniques for tau and amyloid [38] detection such as diffusion tensor imaging (DTI) might not only enable early diagnosis of CTE, but also contribute to the interventions for the prevention of late-onset neurodegenerative diseases following TBI. Studies should be conducted to determine if the brain protein, tau (which could now be measured using blood samples) [39] can be used to detect important SHH events. Results from detailed neuropsychological assessments prior to imaging including mental state examination [40], cognitive assessment [41], non-verbal and letter and category fluency subtests [42], memory abilities [43] [44], executive function system [45], and perception battery [46] should be corroborated with laboratory and imaging tests to define the clinical presentation of the pathology as well. If such findings can be characterized and described thoroughly, ongoing and new investigations will provide a powerful database for studying how SHH results in short- and long-term problems with memory and thinking; and can be used to determine the effectiveness of treatments aimed at preventing these conditions.

## 3. Conclusion and Directions for Future Investigations

**Figure 1.**
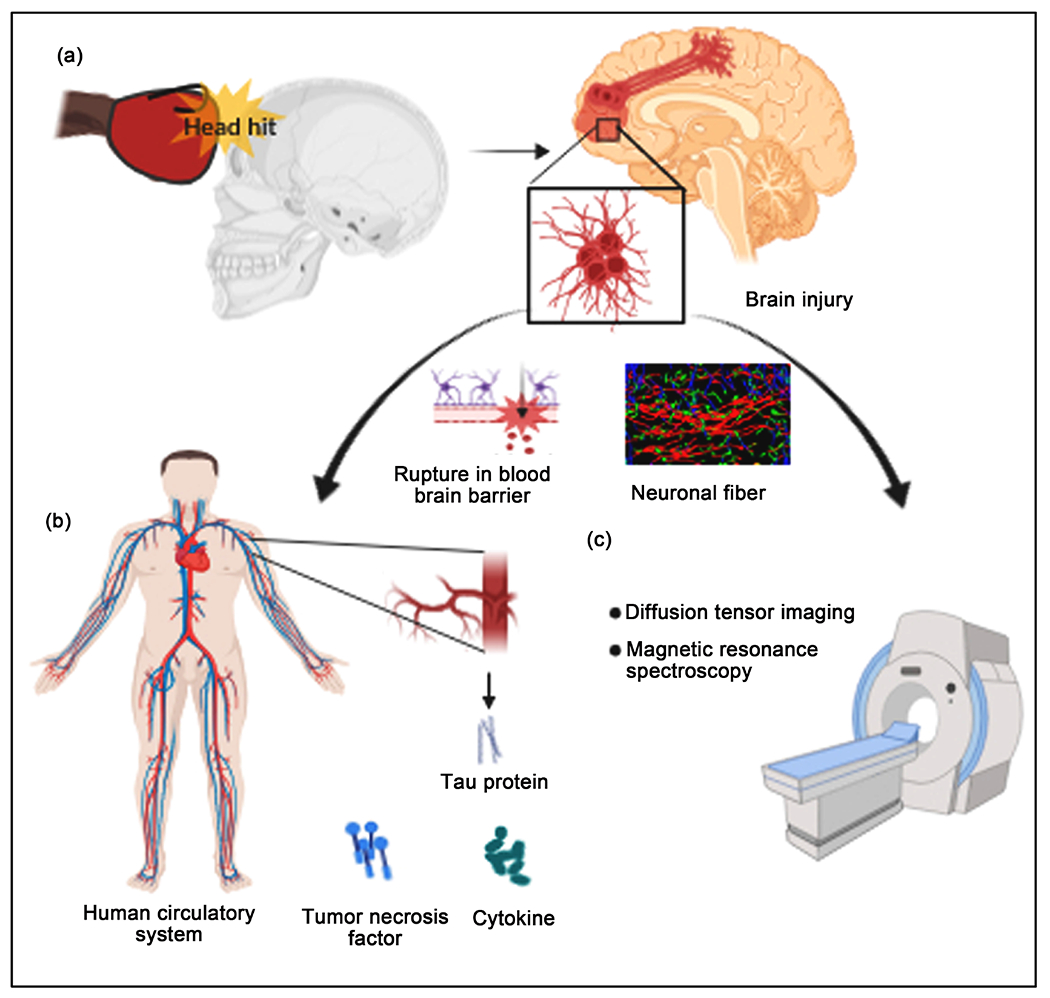
Brain injury in contact sports leads to the release of inflammatory factors in blood and degeneration of neuronal fibers in the human brain that can serve as plausible potential biomarkers for the diagnosis of chronic traumatic encephalopathy. (a) The subject receives a head hit on the skull with a strong force during combat sports (left panel) and the head hit causes damage to the neurons (right panel). The inset figure captures inflamed neuronal fibers highlighted with red color. (b) In case of chronic traumatic encephalopathy, abnormal changes in the permeability of the blood-brain barrier could play a key role in inflammatory responses. Soluble proteins like tau and inflammatory molecules such as cytokine, and tumor necrosis factors get expressed and released in the blood of the human circulatory system. (c) Changes in neuronal fibers could be identified in subjects with chronic traumatic brain injury using magnetic resonance spectroscopy and diffusion tensor imaging.

Boxing and other contact sports are popular worldwide, and more research is required to reduce potential life-debilitating events for at-risk populations of athletes. Training of boxers starts at a very young age and careful monitoring via BBBMs and imaging technologies such as DTI and resting state fMRI to characterize the potential onset of CTEs will be crucial to modifying the training regimes of vulnerable young boxers. Research in this area will lead to the development of interventions, by correlating pharmacotherapy with the pathological onsets in the brain, and associated cognitive deficits in behavior. A multi-scale approach combining molecular and imaging technologies will be the best approach to establish diagnostic and prognostic markers of CTE in vulnerable populations, which is expected to increase over the coming years. Investigations are needed to show that increases in tau are related to SHH, supported by imaging and laboratory studies that can substantiate the physiological manifestations of the molecular changes (Figure 1). Such outcomes can be taken further to design training modules that can compensate for the increase in tau and tissue changes on DTI. Correlation of the increases in tau and changes in brain white matter (WM) or tissue properties such as fractional anisotropy, diffusivity, and as well as behavioral scores will support the emerging concept that SHH can result in subclinical traumatic AI leading to CTE. Demonstrating that tau levels can act as a marker in the detection of SHH and SHH’s role in inducing CTE would allow it to be employed as a unique tool for bridging our understanding of how repetitive head hits may result in short- and long-term neurologic sequelae in athletes in different age-groups. Tau protein could be used for therapeutic studies aimed at mitigating these sequelae. Findings from such investigations could 1) enable healthcare personnel to understand the biomarkers of SHH and prevent additional brain damage after head injury as detected; 2) expand research in other combat sports for example Judo (and other martial arts) that replicate a wide range of pathologies found in athletes with CTE; 3) define molecular and cellular characteristics of brain injury; 4) develop better methods of preventing deterioration of the brain immediately after injury; 5) evaluate and improve therapeutic interventions, and restore function in athletes with a disability. Development of prevention models is necessary to design study trials to estimate the reduction of the frequency and severity of the head injury, including changing behaviors and emphasizing prevention strategies (e.g., improvement and use of protective gears); epidemiological investigations to determine incidence and prevalence of SHH related pathology leading to CTE, and to identify high-risk populations (professional vs. amateur sporting events) and risk factors (age, sex, chronicity and duration). Results from such studies will also advance knowledge at interpreting increases in BBBM in the context of physiological insults from imaging studies in various age cohorts and levels of training (head hits or blast exposure occurring with boxers). Overall, there are gaps in knowledge pertaining to the impact of SHH, its relation to the development of CTE long-term, and further study is needed.

## Abbreviations

CTE: Chronic Traumatic Encephalopathy
SHH: Sub-Concussive Head Hits
AI: Axonal Injury
BBBM: Blood-Based Brain Biomarkers
TBI: Traumatic Brain Injury
WM: White Matter
MRI: Functional Magnetic Resonance Imaging
MRS: Magnetic Resonance Spectroscopy
DTI: Diffusion Tensor Imaging

## References

[1] Jordan BD (1993) Chronic Neurologic Injuries in Boxing. Medical Aspects of Boxing, 177, 185.

[2] Erlanger DM (2015) Exposure to Sub-Concussive Head Injury in Boxing and Other Sports. Brain Injury, 29, 171–174. 10.3109/02699052.2014.965211 25313457

[3] Johnson B, Neuberger T, Gay M, Hallett M and Slobounov S (2014) Effects of Subconcussive Head Trauma on the Default Mode Network of the Brain. Journal of Neurotrauma, 31, 1907–1913. 10.1089/neu.2014.3415 25010992 PMC4238241

[4] Gardner RC and Yaffe K (2015) Epidemiology of Mild Traumatic Brain Injury and Neurodegenerative Disease. Molecular and Cellular Neuroscience, 66, 75–80. 10.1016/j.mcn.2015.03.001 25748121 PMC4461453

[5] Powell JW (2001) Cerebral Concussion: Causes, Effects, and Risks in Sports. Journal of Athletic Training, 36, 307–311. 12937501 PMC155423

[6] Gurdjian ES and Voris HC (1966) Report of ad hoc Committee to Study Head Injury Nomenclature. Neurosurgery, 12, 386–394. 10.1093/neurosurgery/12.CN_suppl_1.386

[7] Cantu RC, (1986) Guidelines for Return to Contact Sports after a Cerebral Concussion. The Physician and Sportsmedicine, 14, 75–83. 10.1080/00913847.1986.11709197 27432133

[8] Poole VN, (2015) Sub-Concussive Hit Characteristics Predict Deviant Brain Metabolism in Football Athletes. Developmental Neuropsychology, 40, 12–17. 10.1080/87565641.2014.984810 25649774

[9] Poole VN, (2014) MR Spectroscopic Evidence of Brain Injury in the Non-Diagnosed Collision Sport Athlete. Developmental Neuropsychology, 39, 459–473. 10.1080/87565641.2014.940619 25144258

[10] Crisco JJ, (2011) Head Impact Exposure in Collegiate Football Players. Journal of Biomechanics, 44, 2673–2678. 10.1016/j.jbiomech.2011.08.003 21872862 PMC3189296

[11] May KH, (2014) Pediatric Sports Specific Return to Play Guidelines Following Concussion. International Journal of Sports Physical Therapy, 9, 242–255. 24790785 PMC4004129

[12] Goldstein M. (1990) Traumatic Brain Injury: A Silent Epidemic. Annals of Neurology, 27, 327. 10.1002/ana.410270315 2327740

[13] Huber BR, (2016) Potential Long-Term Consequences of Concussive and Subconcussive Injury. Physical Medicine and Rehabilitation Clinics of North America, 27, 503–511. 10.1016/j.pmr.2015.12.007 27154859 PMC4866819

[14] Bernick C and Banks S (2013) What Boxing Tells Us about Repetitive Head Trauma and the Brain. Alzheimer’s Research & Therapy, 5, Article No. 23. 10.1186/alzrt177 PMC3706825 23731821

[15] McCrory P, Zazryn T and Cameron P (2007) The Evidence for Chronic Traumatic Encephalopathy in Boxing. Sports Medicine, 37, 467–476. 10.2165/00007256-200737060-00001 17503873

[16] Puvenna V, (2014) Significance of Ubiquitin Carboxy-Terminal Hydrolase L1 Elevations in Athletes after Sub-Concussive Head Hits. PLOS ONE, 9, e96296. 10.1371/journal.pone.0096296 24806476 PMC4012998

[17] Roberts AH (1969) Brain Damage in Boxers: A Study of the Prevalence of Traumatic Encephalopathy among Ex-Professional Boxers. Pitman Medical & Scientific Publishing Co., Ltd., London.

[18] Takahata K, Tabuchi H and Mimura M (2016) Late-Onset Neurodegenerative Diseases Following Traumatic Brain Injury: Chronic Traumatic Encephalopathy (CTE) and Alzheimer’s Disease Secondary to TBI (AD-TBI). Brain and Nerve, 68, 849–857. 10.11477/mf.1416200517 27395469

[19] Jordan BD (2013) The Clinical Spectrum of Sport-Related Traumatic Brain Injury. Nature Reviews Neurology, 9, 222–230. 10.1038/nrneurol.2013.33 23478462

[20] Stern RA, (2011) Long-Term Consequences of Repetitive Brain Trauma: Chronic Traumatic Encephalopathy. PM&R, 3, S460–S467. 10.1016/j.pmri.2011.08.008 22035690

[21] Montenigro PH, (2014) Clinical Subtypes of Chronic Traumatic Encephalopathy: Literature Review and Proposed Research Diagnostic Criteria for Traumatic Encephalopathy Syndrome. Alzheimer’s Research & Therapy, 6, Article No. 68. 10.1186/s13195-014-0068-z PMC4288217 25580160

[22] Mez J, Stern RA and McKee AC (2013) Chronic Traumatic Encephalopathy: Where Are We and Where Are We Going? Current Neurology and Neuroscience Reports, 13, Article No. 407. 10.1007/s11910-013-0407-7 PMC4550089 24136455

[23] Kanner AA, (2003) Serum S100β: A Noninvasive Marker of Blood-Brain Barrier Function and Brain Lesions. Cancer: Interdisciplinary International Journal of the American Cancer Society, 97, 2806–2813. 10.1002/cncr.11409 PMC4135471 12767094

[24] Zetterberg H, Smith DH and Blennow K (2013) Biomarkers of Mild Traumatic Brain Injury in Cerebrospinal Fluid and Blood. Nature Reviews Neurology, 9, 201–210. 10.1038/nrneurol.2013.9 23399646 PMC4513656

[25] Anto-Ocrah M, (2017) Blood-Based Biomarkers for the Identification of Sports-Related Concussion. Neurologic Clinics, 35, 473–485. 10.1016/j.ncl.2017.03.008 28673410

[26] Jay S. (2017) Mild Traumatic Brain Injury and Advanced Magnetic Resonance Imaging Techniques. University of Otago, Dunedin.

[27] Johnson B. (2013) The Effects of Subconcussive Head Trauma. In: Slobounov S and Sebastianelli W, Eds., Concussions in Athletics, Springer, New York, 331–344. 10.1007/978-1-4939-0295-8_19

[28] Xia CF, (2013) [^18^F]T807, a Novel Tau Positron Emission Tomography Imaging Agent for Alzheimer’s Disease. Alzheimer’s & Dementia, 9, 666–676. 10.1016/j.jalz.2012.11.008 23411393

[29] Chien DT, (2013) Early Clinical PET Imaging Results with the Novel PHF-Tau Radioligand [F-18]-T807. Journal of Alzheimer’s Disease, 34, 457–468. 10.3233/lAD-122059 23234879

[30] Johnson KA, (2016) Tau Positron Emission Tomographic Imaging in Aging and Early Alzheimer Disease. Annals of Neurology, 79, 110–119. 10.1002/ana.24546 26505746 PMC4738026

[31] Koerte IK, (2012) White Matter Integrity in the Brains of Professional Soccer Players without a Symptomatic Concussion. JAMA Network, 308, 1859–1861. 10.1001/jama.2012.13735 PMC4103415 23150002

[32] Buerger K, (2006) CSF Phosphorylated Tau Protein Correlates with Neocortical Neurofibrillary Pathology in Alzheimer’s Disease. Brain, 129, 3035–3041. 10.1093/brain/awl269 17012293

[33] McKhann GM, (2011) The Diagnosis of Dementia Due to Alzheimer’s Disease: Recommendations from the National Institute on Aging-Alzheimer’s Association Workgroups on Diagnostic Guidelines for Alzheimer’s Disease. Alzheimer’s & Dementia, 7, 263–269. 10.1016/j.jalz.2011.03.005 PMC3312024 21514250

[34] Vatsalya V, (2015) Effects of Varenicline on Neural Correlates of Alcohol Salience in Heavy Drinkers. International Journal of Neuropsychopharmacology, 18, pyv068. 10.1093/ijnp/pyv068 26209857 PMC4675979

[35] Vatsalya V, (2015) Effects of Varenicline in Human Laboratory Models for Screening of Pharmacotherapeutics for Alcohol Use Disorder: Pi-003. Clinical Pharmacology & Therapeutics, 97, S21.

[36] Kurlawala Z and Vatsalya V (2016) Heavy Alcohol Drinking Associated Akathisia and Management with Quetiapine XR in Alcohol Dependent Patients. Journal of Addiction, 2016, Article ID: 6028971. 10.1155/2016/6028971 PMC5099459 27847671

[37] McKee AC, (2009) Chronic Traumatic Encephalopathy in Athletes: Progressive Tauopathy after Repetitive Head Injury. Journal of Neuropathology & Experimental Neurology, 68, 709–735. 10.1097/NEN.0b013e3181a9d503 19535999 PMC2945234

[38] Stein TD, (2015) β-Amyloid Deposition in Chronic Traumatic Encephalopathy. Acta Neuropathologica, 130, 21–34. 10.1007/s00401-015-1435-y 25943889 PMC4529056

[39] Chen Z, (2019) Learnings about the Complexity of Extracellular Tau Aid Development of a Blood-Based Screen for Alzheimer’s Disease. Alzheimer’s & Dementia, 15, 487–496. 10.1016/j.jalz.2018.09.010 PMC6476313 30419228

[40] Folstein MF, Robins LN and Helzer JE (1983) The Mini-Mental State Examination. Archives of General Psychiatry, 40, 812–812. 10.1001/archpsyc.1983.01790060110016 6860082

[41] Mioshi E, (2006) The Addenbrooke’s Cognitive Examination Revised (ACE-R): A Brief Cognitive Test Battery for Dementia Screening. International Journal of Geriatric Psychiatry, 21, 1078–1085. 10.1002/gps.1610 16977673

[42] Bozeat S, (2000) Non-Verbal Semantic Impairment in Semantic Dementia. Neuropsychologia, 38, 1207–1215. 10.1016/S0028-3932(00)00034-8 10865096

[43] Wechsler D. (1997) Manual for the Wechsler Adult Intelligence Scale-III. The Psychological Corporation, San Antonio.

[44] Hodges JR and Patterson K (2007) *S*emantic Dementia: A Unique Clinicopathological Syndrome. The Lancet Neurology, 6, 1004–1014. 10.1016/S1474-4422(07)70266-1 17945154

[45] Delis DC, Kaplan E and Kramer JH (2001) Delis Kaplan Executive Function System Examiners Manual. San Antonio, TX: NCS Pearson. 10.1037/t15082-000

[46] Warrington EK and James M (1991) The Visual Object and Space Perception Battery. Thames Valley Test Company, Bury St. Edmunds, UK.

